# Intraductal tubulopapillary neoplasm (ITPN) of the pancreas: a distinct entity among pancreatic tumors

**DOI:** 10.1111/his.14698

**Published:** 2022-05-27

**Authors:** Gaetano Paolino, Irene Esposito, Seung‐Mo Hong, Olca Basturk, Paola Mattiolo, Takuma Kaneko, Nicola Veronese, Aldo Scarpa, Volkan Adsay, Claudio Luchini

**Affiliations:** ^1^ Department of Diagnostics and Public Health, Section of Pathology University and Hospital Trust of Verona Verona Italy; ^2^ Institute of Pathology University Hospital of Duesseldorf Duesseldorf Germany; ^3^ Department of Pathology, Asan Medical Center University of Ulsan College of Medicine Seoul South Korea; ^4^ Department of Pathology Memorial Sloan Kettering Cancer Center New York NY USA; ^5^ Department of Hepato‐Biliary‐Pancreatic Medicine NTT Medical Center Tokyo Japan; ^6^ Department of Internal Medicine University of Palermo Palermo Italy; ^7^ ARC‐Net Research Center University and Hospital Trust of Verona Verona Italy; ^8^ Department of Pathology Koç University Hospital and Koç University Research Center for Translational Medicine (KUTTAM) Istanbul Turkey

**Keywords:** intraductal, tubulopapillary, ITPN, IPMN, pancreatic ductal adenocarcinoma, PDAC, pancreas

## Abstract

**Aims:**

Intraductal tubulopapillary neoplasm (ITPN) of the pancreas is a recently recognized pancreatic tumor entity. Here we aimed to determine the most important features with a systematic review coupled with an integrated statistical approach.

**Methods and results:**

PubMed, SCOPUS, and Embase were searched for studies reporting data on pancreatic ITPN. The clinicopathological, immunohistochemical, and molecular data were summarized. Then a comprehensive survival analysis and a comparative analysis of the molecular alterations of ITPN with those of pancreatic ductal adenocarcinoma (PDAC) and intraductal papillary mucinous neoplasm (IPMN) from reference cohorts (including the International Cancer Genome Consortium‐ ICGC dataset and The Cancer Genome Atlas, TCGA program) were conducted. The core findings of 128 patients were as follows: (i) Clinicopathological parameters: pancreatic head is the most common site; presence of an associated adenocarcinoma was reported in 60% of cases, but with rare nodal metastasis. (ii) Immunohistochemistry: MUC1 (>90%) and MUC6 (70%) were the most frequently expressed mucins. ITPN lacked the intestinal marker MUC2; unlike IPMN, it did not express MUC5AC. (iii) Molecular landscape: Compared with PDAC/IPMN, the classic pancreatic drivers *KRAS*, *TP53*, *CDKN2A*, *SMAD4*, *GNAS*, and *RNF43* were less altered in ITPN (*P* < 0.001), whereas *MCL* amplifications, *FGFR2* fusions, and *PI3KCA* mutations were commonly altered (*P* < 0.001). (iv) Survival analysis: ITPN with a “pure” branch duct involvement showed the lowest risk of recurrence.

**Conclusion:**

ITPN is a distinct pancreatic neoplasm with specific clinicopathological and molecular characteristics. Its recognition is fundamental for its clinical/prognostic implications and for the enrichment of potential targets for precision oncology.

## Graphical abstract

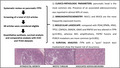

## Introduction

Pancreatic ductal adenocarcinoma (PDAC) is a highly malignant neoplasm responsible for the vast majority of pancreatic tumor deaths.^1^, ^2^ The high mortality rate is due to its biological aggressiveness and the difficulties in early diagnosis, which remains a major challenge in the era of personalized medicine.^1^, ^2^ Along this line, a key factor for addressing this issue is the study of precursor lesions, which can improve the understanding of the biology of pancreatic cancer as well as the strategies for the early diagnosis and treatment of this condition.^3^, ^4^, ^5^

The current Word Health Organization (WHO) classification of tumors of the digestive system recognized five types of potential PDAC precursors: pancreatic intraepithelial neoplasia (PanIN), mucinous cystic neoplasm (MCN), intraductal papillary mucinous neoplasm (IPMN), intraductal oncocytic papillary neoplasm (IOPN), and intraductal tubulopapillary neoplasm (ITPN).^6^

Notably, pancreatic ITPN represents the latest addition to the intraductal subgroup of lesions and was introduced in the 2010 edition of the WHO classification.^7^ It accounts for up to 3% of all intraductal pancreatic neoplasms and shows distinct and peculiar features.^6^ Initially, it was reported under the “intraductal tubular adenocarcinomas” category and was strictly defined as being exclusively tubular,^6^ but the name was eventually changed to “intraductal tubulopapillary.” This term is widely used in current practice.^6^ These definitional differences have led to different perspectives regarding the nature of these tumors. Regardless, the entity is gaining increasing research interest owing to its prognostic implications and the potential presence of molecular alterations that are responsive to targeted therapies.^5^

**Figure 1:**
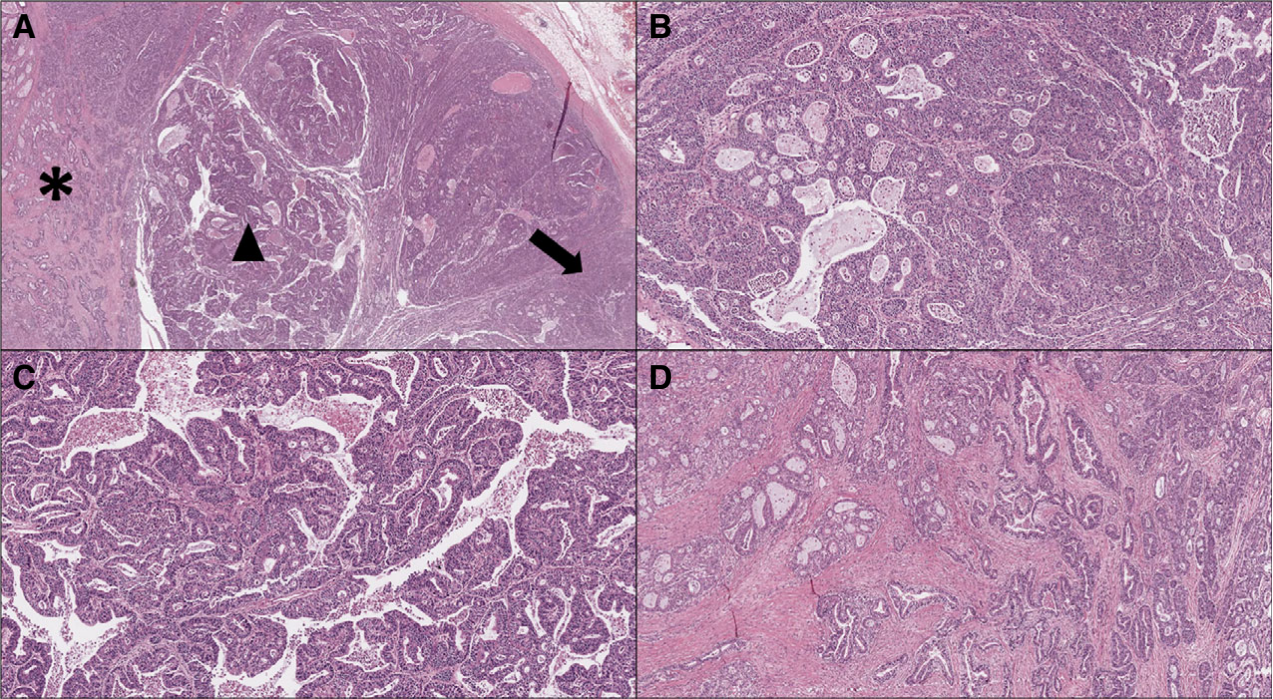
Typical histology of pancreatic ITPN with an associated invasive adenocarcinoma. (**A**) Low‐magnification image for appreciating the different types of architecture that can be encountered in ITPN: the black arrow indicates the tubular architecture, which is generally predominant, the black triangle indicates the papillary component, which is not a constant presence in this type of lesion, and the asterisk indicates the infiltrative component (hematoxylin–eosin, 4× original magnification). (**B,C,D**) Higher magnification of the tubular (**B**), of the papillary (**C**), and of the infiltrative (**D**) components (hematoxylin–eosin, 10 × original magnification). [Colour figure can be viewed at wileyonlinelibrary.com]

**Figure 2:**
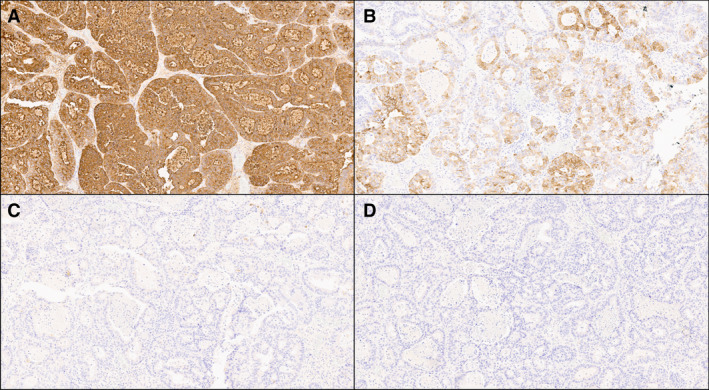
Classical immunohistochemical patterns of mucins expression in pancreatic ITPN (10× original magnification). (**A**) MUC1: this is the mucin more often expressed by pancreatic ITPN. (**B**) MUC6: this is another mucin very often expressed by pancreatic ITPN, sometimes it appears more focal. (**C**) MUC5AC: it is usually negative in ITPN, different from IPMN. (**D**) MUC2: it is usually negative in ITPN, different from intestinal IPMN. [Colour figure can be viewed at wileyonlinelibrary.com]

Histologically, ITPN is composed almost exclusively of tubular glands arranged in a back‐to‐back fashion, with irregular papillary structures and low‐to‐null mucin production.^3^, ^4^, ^5^, ^6^ High‐grade dysplasia is usually homogeneously distributed within the lesion (Figure 1). From an immunohistochemical point of view, ITPN usually exhibits a distinct profile based on the pattern of mucin expression, with positivity for MUC1 and MUC6 and a low level of MUC2 and MUC5AC expression^4^, ^5^, ^6^ (Figure 2). Although its morphology represents the most important diagnostic criterion, this specific staining pattern can be used as a basis for establishing the cell lineage, thus supporting the differential diagnosis of IPMN and IOPN.

Given its relatively recent introduction as a tumor entity, its rarity, and the shifting criteria in its definition over the years, ITPN still remains an underrecognized category. Furthermore, very few studies have described case series based on a substantial number of cases, with the vast majority of ITPN cases described in a single case report. This systematic review, coupled with an integrated statistical approach, aimed to summarize and clarify the specific histological, immunohistochemical, and molecular features of this tumor entity, with a specific focus on these parameters in terms of the potential implications on survival indices, and to provide practical information to guide the management of ITPN, from routine diagnostic tests to clinical treatment.

## Materials and methods

This systematic review was conducted without language restrictions and adhered to the Meta‐analysis of Observational Studies in Epidemiology guidelines and Preferred Reporting Items for Systematic reviews and Meta‐Analyses statement^8^, ^9^ following a predetermined unpublished protocol. The protocol of the review is available by the corresponding author upon request.

### Inclusion and exclusion criteria

(i) Original studies evaluating human ITPN, (ii) studies that documented the pancreatic origin of the neoplasm, (iii) studies that reported the undebatable presence of the histological demonstration of ITPN, and (iv) studies that were published in a peer‐reviewed journal were included in this systemic review. By contrast, (a) studies that used preclinical or mouse models of ITPN, (b) studies that did not report the pancreatic origin of the neoplasm (exclusion of the biliary counterpart), and (c) studies that only used preliminary data from published abstracts. Case reports on intraductal pancreatic neoplasms, described before the official recognition of ITPN as a specific tumor entity, were also reviewed and included if they satisfied the ITPN diagnostic criteria according to the WHO classification.^6^

### Data sources and literature search strategy

Two investigators (G.P. and C.L.) independently searched PubMed, SCOPUS, and Embase up to May 31, 2021. The terms used in searching PubMed included combinations of the following keywords: (“intraductal tubulopapillary neoplasm*” OR “intraductal tubule‐papillary neoplasm*” OR “ITPN” OR “intraductal tubular” OR “tubulopap*” OR “tubulo‐pap*”) AND (“pancreatic” OR “pancreas” OR “pancrea*”). Similar terms were used to find related articles in SCOPUS and Embase databases. The reference lists of all included articles and previous related reviews were also searched for relevant studies.

### Study selection

Following the searches outlined above, after removal of duplicates, two independent reviewers (G.P. and C.L.) screened the titles and abstracts of all potentially eligible articles. The two authors applied the eligibility criteria, reviewed the full texts, and reached a final list of selected articles through consensus with a third author (A.S.). In cases of double/overlapped cohorts, a cohort with a larger amount of available data was selected.

### Data extraction and synthesis

Two authors were involved in the data extraction using a standardized Microsoft Excel database. Specifically, one author (G.P.) extracted the data from the included articles, while another independent author (C.L.) validated the data.

For each article, information about the authors, year of publication, country of origin of the analyzed cohort, number of patients, age and sex of patients, involved pancreatic region, presence of an associated invasive cancer, tumor size, pTNM, presence of vascular invasion and perineural infiltration, R status, type of ductal involvement, main symptoms, main radiologic findings, and survival outcomes were extracted. Finally, all extracted data are presented in Table S1 and summarized in Table 1. A comprehensive statistical analysis was conducted for all clinicopathological variables to identify any potential indicators of high‐risk lesions (e.g. invasive component, tumor size, or nodal metastasis).

**Table 1:** Summarizing table of the most important clinic‐pathologic parameters of pancreatic ITPN

Total number of cases/lesions	Gender	Mean age at diagnosis	Site in the pancreas	Associated cancer	Mean tumor Size	pT^b^	pN^b^	Vascular invasion^b^	Perineural invasion^b^	Involved duct(s)	Symptoms	Main radiologic findings
128/131^a^	Male: 55.5%; Female 45.5%	60.3 years	H: 51.7%; B: 18.6% T: 10.2% ML: 19.5%	Yes: 58.6%; No: 41.4%	38.7 mm (5–150)	T1: 26.3%; T2: 31.6%; T3: 42.1%, T4: 0%	N0: 74.2%; N1: 22.6%; N2: 3.2%	Yes: 81.8%; No: 18.2%	Yes: 20%; No: 80%	M: 72.5%; BR: 17.5%; MX: 10%	Abdominal pain (38.2%); no symptoms (31.7%); jaundice (5.7%), diarrhea (3.2%)	S: 61.3%, SC: 21.0%, C: 17.7%

H, head; B, body; T, tail; ML: involvement of multiple pancreatic regions; pT: pathological tumor stage; pN: pathological nodal stage; M, main duct; BR, branch duct; MX, mixed; C, radiologic cystic appearance; S, radiologic solid/mass‐forming lesion; SC, radiologic mixed cystic‐solid appearance.

^a^ In three patients also data for local disease recurrence have been reported, thus the overall number of patients is 128, whereas the overall number of described pancreatic ITPN is 131.

^b^ The data was from IPTN cases with associated cancers and available information of pT (*n* = 19), pN (*n* = 31), vascular (*n* = 11), and perineural (*n* = 5) invasion.

After collecting the clinicopathologic data, all information regarding the immunohistochemical expression patterns of the mucins most commonly used in daily diagnostic practice, namely, MUC1, MUC2, MUC5AC, and MUC6, are listed in Table S2 and summarized in Table 2.

**Table 2:** Summarizing table of mucins expression in pancreatic ITPN

Mucins	MUC1	MUC2	MUC5AC	MUC6
% Overall positivity	90.4%	2.7%	8.6%	69.8%
% Lack of expression	9.6%	97.3%	91.4%	30.2%

The data were retrieved from available information on mucin expression in pancreatic ITPN: MUC1 (*n* = 73), MUC2 (*n* = 76), MUC5AC (*n* = 80), and MUC6 (*n* = 63).

### Molecular analysis

Finally, the information regarding the genetic alterations of all reported ITPN cases is also reported in Table S3 and summarized in Table 3, which highlights the molecular differences of ITPN with PDAC and IPMN. All genetic alterations were also investigated by conducting a pathway analysis using ShinyGO (http://bioinformatics.sdstate.edu/go/), based on all available gene sets and with a cutoff *P*‐value of 0.05. The gene set of our cases was tested for pathway clustering and in relation to the major pathways of other cancer types, including pancreatic cancer. The collected clinicopathological, immunohistochemical, and molecular data were then analyzed, interpreted, and discussed by all authors.

**Table 3:** Summarizing table of the most distinguishing molecular alterations of pancreatic ITPN vs. PDAC and IPMN

Altered gene	Frequency in ITPN	Difference with PDAC/IPMN
*KRAS* mutations	10.4%	More common in PDAC/IPMN, *P* < 0.001
*TP53* mutations	4.7%	More common in PDAC/IPMN, *P* < 0.001
*CDKN2A* mutations	27.2%	More common in PDAC/IPMN, *P* < 0.001
*SMAD4* mutations	0%	More common in PDAC/IPMN, *P* < 0.001
*GNAS* mutations	0%	More common in PDAC/IPMN, *P* < 0.001
*RNF43* mutations	0%	More common in PDAC/IPMN, *P* < 0.001
*MCL* amplifications	31.8%	More common in ITPN, *P* < 0.001
*FGFR2* fusions	18.2%	More common in ITPN, *P* < 0.001
*PI3KCA* mutations	13.6%	More common in ITPN, *P* < 0.001

PDAC, conventional pancreatic ductal adenocarcinoma; IPMN, intraductal papillary mucinous neoplasm.

To definitively assess the differences in molecular features between conventional PDAC and ITPN, the Fisher's exact test was used to compare the ITPN genetic alterations with the PDAC/IPMN molecular data published by the International Cancer Genome Consortium (https://www.icgc.org, last access 06/30/2021) and The Cancer Genome Atlas Research Network (TCGA),^10^ which were used as reference cohorts. *P* ≤ 0.05 was considered significant.

### Survival analysis

The association between clinicopathological, immunohistochemical, and molecular variables and survival indices was explored using Cox's regression analysis and graphically reported using Kaplan–Meier curves if statistically significant. The results were calculated as hazard ratios (HRs) with the corresponding 95% confidence intervals (CIs). *P* ≤ 0.05 was considered significant.

## Results

The literature search identified 213 unduplicated articles, 74 of which were excluded after reviewing their titles and abstracts; hence, only 139 articles were eligible for full‐text review. After applying our inclusion criteria, 68 articles were included in the systematic review (Figure S1).^11^, ^12^, ^13^, ^14^, ^15^, ^16^, ^17^, ^18^, ^19^, ^20^, ^21^, ^22^, ^23^, ^24^, ^25^, ^26^, ^27^, ^28^, ^29^, ^30^, ^31^, ^32^, ^33^, ^34^, ^35^, ^36^, ^37^, ^38^, ^39^, ^40^, ^41^, ^42^, ^43^, ^44^, ^45^, ^46^, ^47^, ^48^, ^49^, ^50^, ^51^, ^52^, ^53^, ^54^, ^55^, ^56^, ^57^, ^58^, ^59^, ^60^, ^61^, ^62^, ^63^, ^64^, ^65^, ^66^, ^67^, ^68^, ^69^, ^70^, ^71^, ^72^, ^73^, ^74^, ^75^, ^76^, ^77^, ^78^

### Clinicopathologic features

The clinicopathological features are presented in Table 1. Overall, the entire cohort was composed of 128 patients with a total of 131 ITPN patients (three patients experienced pancreatic relapse, which was included in the systematic review). Data regarding sex were available in 126 patients; the proportion of male patients (70/126, 55.5%) was slightly higher than that of female patients (56/126, 44.5%). The patient's mean age at the time of diagnosis was 60.3 years (range: 34–85). Data regarding patient's origin were available in 96 patients; the proportion of patients from Asia (77/96, 80.2%) was relatively high compared with that from other continents. Tumor location was specified in 118 patients.

The most common site was the pancreatic head (61/118, 51.7%), followed by the body (22/118, 18.6%) and tail (12/118, 10.2%). Patterns of a larger extension in the pancreatic gland were represented by diffuse involvement of the head, body, and tail (12/118, 10.2%), followed by the body‐tail (7/118, 5.9%) and head‐body (4/118, 3.4%). The presence of an invasive component was reported in 68 of the 116 (58.6%) patients. Data on tumor size (entire lesion) were available in 104 patients, and the mean size was 38.7 mm (range: 5–150 mm). Most studies did not separately indicate the size of invasive carcinoma. As regards the pathologic TNM classification for ITPN presenting an invasive component, the distributions, where available, were as follows: (i) tumor stage: pT1, 5/19, 26.3%; pT2, 6/19, 31.6%; pT3, 8/19, 42.1%; and pT4, 0/19, 0%. The pTNM stage was provided based on the current 8th edition of the American Joint Committee on Cancer staging system^79^ when the information was available in the article. However, in most studies the stage was based on the overall size of the tumor rather than the invasive component, which is the approach recommended in consensus articles^80^; (ii) nodal stage: pN0: 23/31, 74.2%; pN1: 7/31, 22.6%; and pN2: 1/31, 3.2%; and (iii) distant metastasis stage: M0, 28/29, 96.6% and M1, 1/29, 3.4%. For lesions with an infiltrative component, vascular invasion was assessed in 11 patients and reported in 9 of 11 (81.8%) patients; meanwhile, perineural invasion was reported in one out of five (20%) patients. Margin status (pathologic R stage) was documented for 27 tumors, with the majority of the patients having R0 stage (25/27, 92.6%) and two having R1 stage (2/27, 7,4%). With regard to pancreatic ductal tree involvement, these data were obtained for 80 tumors. ITPN more often involved the main pancreatic/Wirsung duct (58/80, 72.5%), followed by the branch ducts (14/80, 17.5%) and mixed patterns, the latter indicating the involvement of both main and branch ducts (8/80, 10%).

The most common general symptom was abdominal pain (47/123, 38.2%), followed by jaundice (7/123, 5.7%) and diarrhea (4/123, 3.2%). Other less common symptoms included pancreatitis, weight loss, and exacerbation of diabetes mellitus, the latter associated with a diffuse pattern of pancreatic involvement (2/2 cases). A remarkable number of patients (39, 31.7%) did not develop any symptoms, of whom 15 (38.5%) had a noninvasive lesion, 16 (41.0%) had an invasive lesion, and the remaining eight (8/39, 20.5%) did not specify the type of lesion.

At imaging, the majority of lesions were solid masses (38/62, 61.3%), followed by solid/cystic (13/62, 21.0%) and cystic (11/62, 17.7%). None of the clinicopathological features were significantly associated with the presence of high‐risk parameters; however, a positive trend was observed between ITPN with ≥40 mm tumor size and the presence of an associated infiltrative adenocarcinoma component (*P* = 0.10, Fisher's exact test).

### Immunohistochemistry and molecular profiles

With regard to the results of immunohistochemistry, the following mucins were most commonly expressed: MUC1, the most common type (66/73 cases, 90.4% of overall positivity), and MUC6 (44/63 cases, 69.8%). The other two mucins were not expressed in ITPN: MUC2 was negative in 97.3% (73/75) of patients, while MUC5AC was not expressed in 91.4% (74/81) of the patients (Table 2).

In terms of the molecular profile of ITPN, the prevalence of alterations affecting the five classic PDAC/IPMN driver genes was as follows: *KRAS* mutated in 10.4% (5/48) of the patients, *TP53* mutated in 4.7% (2/43) of the cases, *CDKN2A* was altered in 27.2% (6/22) of the cases, and *SMAD4*, *GNAS*, and *RNF43* were altered in 0% of the cases. Recurrent alterations included *MCL* amplification, observed in 31.8% (7/22) of the cases; *FGFR2* fusions, detected in 18.2% (4/22) of the cases; *PI3KCA* mutations, found in 13.6% (6/44) of the cases; and mutations in *the MLL‐gene* family, reported in 22.7% (5/22) of the patients (Table 3). Pathway analysis demonstrated that PI3K‐Akt was the most activated pathway in patients with pancreatic ITPN; the statistical associations considering all cancer types, based on the pathway analysis, were more significant in breast and hepatocellular cancers than in conventional pancreatic cancer (Figure S2).

Comparison of these molecular findings using the IGCG/TCGA data showed that the differences between ITPN and conventional PDAC (for *KRAS*, *TP53*, *CDKN2A*, and *SMAD4*)/IPMN (for *GNAS* and *RNF43*) were significant. *KRAS*, *TP53*, *CDKN2A*, *SMAD4*, *GNAS*, and *RNF43* were more commonly altered in PDAC/IPMN than in ITPN (*P* < 0.001, Fisher's exact test). With regard to the recurrent ITPN alterations, *MCL* amplifications, *FGFR2* fusions, and *PI3KCA* mutations were significantly more common in ITPN than in PDAC/IPMN (*P* < 0.001, Fisher's exact test) (Table 3).

Regarding the material for molecular analysis, the vast majority of studies were based on formalin‐fixed paraffin‐embedded (FFPE) tissues. No studies performed multiregional sequencing within the same tumor mass, but one study presented data from a primary tumor, its local recurrence, and a matched nodal metastasis.^55^ Lastly, only one study declared the use of laser microdissection from FFPE tissue for molecular analysis.^59^

### Survival analysis

**Figure 3:**
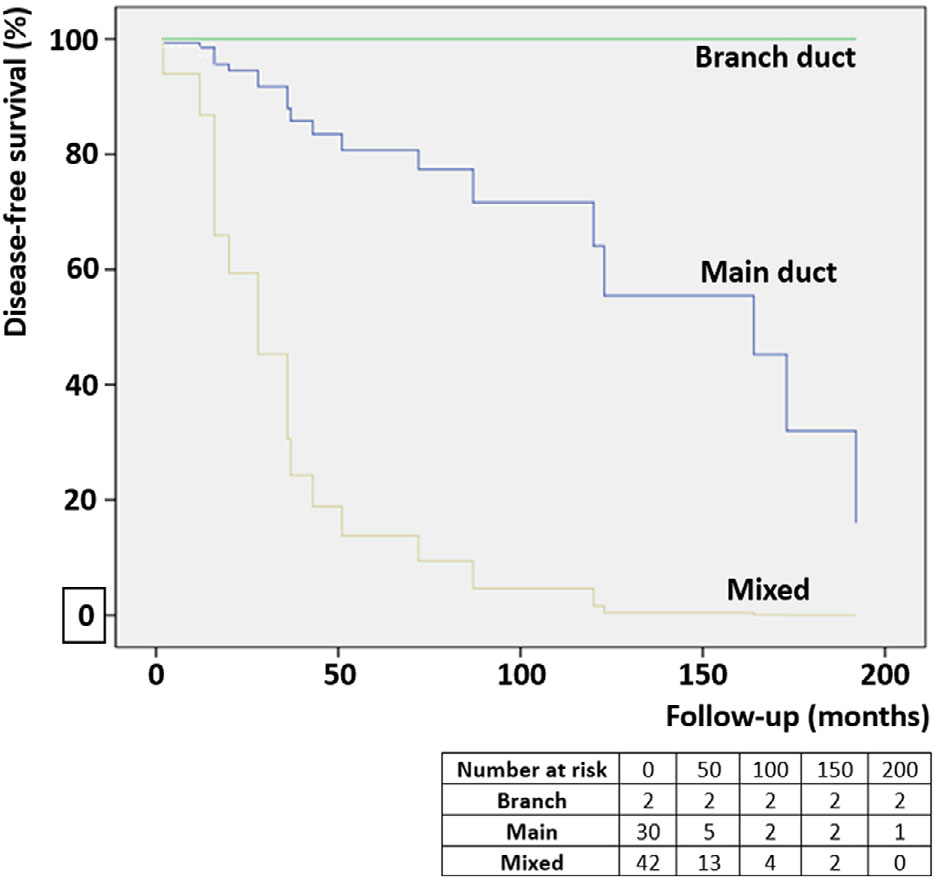
Kaplan–Meier curve regarding disease‐free survival of patients with pancreatic ITPN based on the different pattern of ductal tree involvement. [Colour figure can be viewed at wileyonlinelibrary.com]

Using Cox regression analysis, no significant associations were found between almost all parameters and survival indices. Notably, there was one exception―the type of ductal‐tree involvement. Indeed, mixed‐duct involvement (i.e., main duct and branch duct, simultaneously) was associated with a higher risk of relapse (HR = 9.2, 95% CI: 1.54–55.21, *P* = 0.015) than the main duct (*P* = 0.11) and branch duct (*P* = 0.99) involvement, with the latter showing the lowest risk of recurrence (Figure 3).

## Discussion

In this article, a systematic review of the clinicopathological, immunohistochemical, and molecular features of pancreatic ITPN was conducted, providing statistical analyses for survival and comparing the molecular profiles using the data from existing datasets. The core findings of this study were divided into the following four main areas: (i) clinicopathological parameters, (ii) immunohistochemistry (IHC), (iii) molecular landscape, and (iv) survival analysis.

### Clinicopathologic parameters

**Figure 4:**
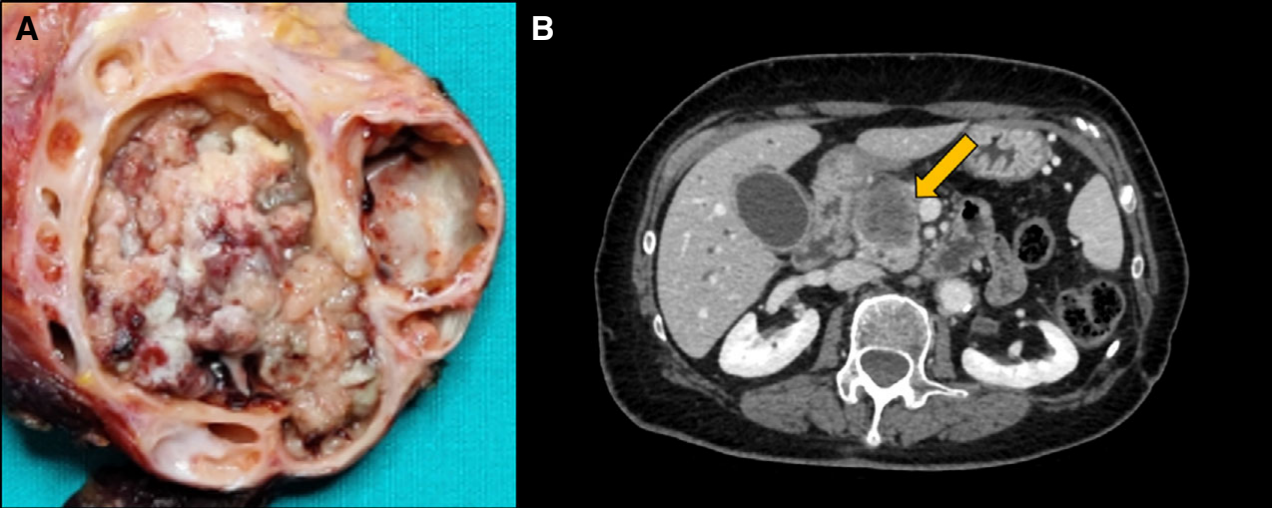
Typical appearance of a case of pancreatic ITPN, showing solid and solid/cystic areas (**A**: solid/cystic appearance at gross sampling, **B**: solid and solid/cystic appearance at imaging: CT scan, where the lesion is indicated by a yellow arrow). [Colour figure can be viewed at wileyonlinelibrary.com]

After analyzing the data from the literature, the following clinicopathologic characteristics of ITPN were highlighted: (i) They tend to form more solid nodular tumors unlike their kindred IPMN, which are mostly cystic (Figure 4); (ii) although they are slightly more common in the head, close to half of the patients showed body/tail involvement, while a third showed left‐sided predominance; (iii) the presence of an invasive component was reported in almost 60% of the patients; however, the invasive component is often small, although in many studies this was not specifically documented; (iv) in the case of an invasive component, vascular invasion (>80%) was a common finding, whereas nodal metastasis (25.8%) and perineural invasion (20%) rarely occur; (v) Wirsung duct (“pure” involvement) was the most commonly involved (72.5%), followed by branch ducts (17.5%) and by mixed pattern (10%); and (vi) at imaging, solid or solid‐cystic aspect was the most common finding (>80%).

The results related to the clinicopathological variables were in line with the data from the most relevant studies on pancreatic ITPN.^23^, ^24^, ^54^, ^55^ Simultaneously, these data highlight some interesting aspects. The first aspect is the high rate (almost 60%) of prevalence of an associated invasive component at the time of diagnosis. This rate is higher than that observed for IPMN,^3^, ^81^, ^82^ highlighting the importance of considering ITPN as a nonnegligible precursor lesion in adenocarcinoma. However, ITPN is a highly complex lesion with frequent retrograde cancerization into the atrophic lobules, which creates a pattern virtually indistinguishable from true invasive carcinomas. Furthermore, the artifactual clefts in the small ductules may create an impression of vascular invasion. These mimicries of pseudoinvasion may partly explain why only 25% of the patients with reported invasion have nodal metastasis and the protracted clinical course of even ITPN that have been classified as “invasive.” Along these lines, most of the T‐stages provided in the literature for these tumors were based on the overall size of the tumor, although consensus recommendations indicated that this should be based on the invasive component.^80^ The low rate of nodal metastasis might also suggest the slow growth of these lesions. Taken together, these findings highlight the importance of considering ITPN as a distinct neoplastic entity among pancreatic tumors and improving the strategies for the early detection of this type of lesion. Finally, the differences with other types of intraductal lesions are also evident in radiology, where a solid or, less frequently, a solid‐cystic mass represents the classic feature, which is different from the predominant cystic appearance of IPMN.^83^

Morphologically (and definitionally), ITPN overlaps with a spectrum of other intraductal neoplasms, which is also highlighted in the definitions and illustrations in the literature. In previous studies, these tumors were defined based on their tubular pattern.^11^, ^17^, ^18^ Accordingly, any intraductal tubular neoplasm, including mucinous pyloric gland, was also considered in this group.^12^, ^13^, ^14^ In a more recent analysis, the absence of overt intracystoplasmic mucin (and lack of MUC5AC) is now regarded as a crucial part of the definition. However, this approach was not uniformly employed. In the earlier literature, a lack of papillae was regarded as a defining characteristic of this entity.^11^ Subsequently, the presence of papillary elements was accepted, and the term ITPN was proposed.^16^, ^23^ This has led to the inclusion of some gastro‐pancreatobiliary‐type IPMNs that can have a prominent tubular pattern that can also be included in the ITPN category, which is evident in some of the current publications. Therefore, minimal papillary structure formation or the absence of papillary structure formation, as well as the absence of intracytoplasmic mucin (and minimal or absence of MUC5AC expression), should be used as a definition for this entity. The main histomorphological differential diagnosis of ITPN is acinar cell carcinoma.^84^

### IHC

Analysis of the reported expression patterns of mucins most commonly used in routine diagnostic activities showed that MUC1 (>90% positivity rate) and MUC6 (almost 70% positivity rate) emerged as the typical ITPN mucins, whereas MUC2 and MUC5AC were rarely expressed.

The expression of MUC1 and MUC6 indicates that ITPN has clear pancreatic ductal differentiation and may also show gastric‐pyloric differentiation.^53^ The very low rate of expression of the pan‐IPMN marker MUC5AC suggests a lack of gastric‐foveolar differentiation and distinguishes this entity from IPMN.^54^ After completing the potential gastroenteric lineage of differentiation, a very low rate of MUC2 expression indicates a lack of intestinal differentiation. Thus, ITPN does not show gastrointestinal differentiation that is commonly observed in the different subtypes of IPMN, and instead shows pancreatic ductal differentiation.^54^ The IHC profile of ITPN based on the mucin expression pattern appears to be specific for this type of lesion and can be of great help in the differential diagnosis during routine diagnostic activity. Indeed, the potential differential diagnoses of ITPN include not only lesions with typical intraductal growth, such as IPMN and IOPN, but also other neoplasms that can only show this pattern occasionally, such as acinar cell carcinoma and well‐differentiated neuroendocrine tumors with intraductal growth.^84^, ^85^ Thus, an IHC panel for the differential diagnosis of pancreatic neoplasms with intraductal growth should always include these different types of mucins along with the markers of acinar and neuroendocrine differentiation. Finally, since the results of IHC can be affected by the specific clone used^86^ and given the heterogeneity of clones used in different studies (Table S2), routine diagnostic activity should be performed following standardized protocols. Along this line, it should be noted that, for pancreatic ITPN, WHO guidelines do not recommend a specific clone for the different mucins/antibodies, and in our comprehensive analysis we did not find a superior or more specific clone. Therefore, regarding MUC1, MUC2, MUC5AC, and MUC6, the most important point seems to be represented by the simultaneous and the correct use of all four of these mucins, and the integration of their expression pattern with tumor morphology. The absence of specific clones further highlights the importance of histology in this relevant task.

### Molecular landscape

By comparing the extracted data on the molecular profiles of ITPN with the existing datasets of pancreatic tumors, the classic pancreatic drivers of PDAC/IPMN, such as *KRAS*, *TP53*, *CDKN2A*, *SMAD4*, *GNAS*, and *RNF43*, were significantly less altered in ITPN than in PDAC/IPMN (*P* < 0.001), whereas the *MCL* amplifications, *FGFR2* fusions, and *PI3KCA* mutations were significantly more common in ITPN than in PDAC/IPMN (*P* < 0.001).

The diversity of the genetic landscape between ITPN and PDAC/IPMN was confirmed at the molecular level based on the morphological and immunohistochemical peculiarities of this type of lesion and further indicates that ITPN represents a distinct clinicopathologic entity in the pancreas.^55^ Notably, these findings may also have potential implications for targeted therapeutic strategies. Along this line, *MCL* could be considered as a target of various treatments and is already under evaluation in clinical trials (e.g. NCT04178902, NCT02992483). Preclinical evidence has shown encouraging outcomes in hematologic malignancies, including acute myelogenous leukemia, multiple myeloma, and solid cancers.^87^, ^88^ The rate of *PI3KCA* gene mutations^24^, ^33^, ^55^ and that of other factors of the phosphatidylinositol‐pathway, such as *PIK3CB*, *INPP4A*, and *PTEN*, were higher in ITPN than that in PDAC/IPMN.^55^ Notably, pathway analysis showed that PI3K‐Akt was the most activated pathway in pancreatic ITPN. This finding is of particular interest for expanding the therapeutic opportunities in patients with ITPN, representing some of the alterations of such pathway potential actionable targets.^89^, ^90^ Moreover, *FGFR2* fusions were recently detected in ITPN. Basturk *et al*. described four patients with this type of alteration,^55^ which appeared to be enriched in ITPN compared with that in PDAC/IPMN. Since there is a growing body of evidence regarding the sensitivity of neoplastic cells harboring such fusions to FGFR inhibitors,^91^, ^92^ this alteration should be taken into account during the molecular diagnosis of ITPN and when developing tailored therapeutic approaches. A final consideration of the genetic landscape of pancreatic ITPN should be reserved for chromatin remodelers, such as *MLL genes* and *BAP1*, which seemed to be more altered in this type of lesion compared with that in conventional PDAC (although the comparison with existing datasets did not show statistical significance). Notably, a recent study on the biliary counterpart of ITPN, based on whole‐exome sequencing, confirmed the important role played by this class of genes in the whole ITPN spectrum.^93^ In addition to mutations in chromatin remodelers, other correspondences of pancreatic ITPN with the biliary counterparts, further highlighting their similarities, included the common IHC profile and frequent chromosome 1q gains.^93^

### Survival analysis

Summarizing the data on patient survival remained challenging due to the issues derived from original studies, with several studies having short follow‐up periods. This may be one of the most important reasons for the lack of significant associations between clinicopathological parameters and survival outcomes. In this scenario, it is important to highlight that, in the cohort of Basturk *et al*., significant differences were found in the OS between ITPN alone and ITPN with an associated invasive adenocarcinoma and conventional PDAC.^54^ This difference was not as evident as that reported in the whole cohort of published ITPN studies; however, this was most likely due to the highly variable definitions in the literature; in this study,^54^ strict criteria currently indicated in the most recent WHO guidelines and consensus articles were employed.

Our comprehensive survival analysis also identified one parameter that was not previously recognized as a potentially significant prognostic indicator: the pattern of ductal tree involvement. Indeed, mixed‐duct involvement was associated with a higher risk of ITPN relapse (HR = 9.2, *P* = 0.015) than the main duct (*P* = 0.11) and branch duct (*P* = 0.99) involvement, with the latter showing the lowest risk of recurrence. Although this classification was frequently used as a radiological classification, such data were retrieved from articles that histologically described ductal tree involvement. The results regarding the “pure” branch duct involvement of ITPN were in line with the well‐established knowledge of branch‐duct IPMN, which is the IPMN subtype with the lowest malignant potential.^94^ In ITPN, main duct involvement (mixed or “pure”) was a risk factor for disease relapse, similar to IPMN; in IPMN, this finding is true for main duct lesions and if the duct diameter is >10 mm.^95^ The implications derived from the modality of ductal tree involvement, together with intraductal growth, represent one of the very few contact points between ITPN and IPMN and may indicate the potential importance of correct gross sampling for the identification of the type of ductal involvement.

The findings of this study highlight the following issues regarding ITPN, which are currently underappreciated. ITPN should be considered in the differential diagnosis when an intraductal tumor shows more nodular solid growth patterns. The paucity of studies focusing on the characteristics of this entity on fine‐needle aspiration (FNA) biopsy indicates that it is not yet fully recognized in this field. Considering that most pancreatic tumors are initially diagnosed with FNA, it is possible that this underrecognized entity is often misdiagnosed during FNA. In surgical pathology, because invasiveness is commonly reported in ITPN, the total sampling of these tumors may be more crucial than that in other intraductal neoplasms. On the contrary, it appears that in reporting and staging these tumors, most studies did not separate the invasive component; the size of the invasive component ought to be provided separately, and T‐staging was determined based on this invasive component. Molecular analysis is advisable for this peculiar tumor type, since it would both provide confirmatory information as well as potentially lead to targetable pathways, especially considering that most are curable tumors but may ultimately lead to mortality. Genomic profiling to detect the potential targets for precision oncology may be useful in unresectable and/or metastatic tumors detected at the time of diagnosis.

Our study has some limitations. First, the majority of data were obtained from single case reports or small case series, thus reducing their reliability in the comparative analysis. Furthermore, the survival analysis was based on a relatively small sample size. Although the modality of ductal involvement appeared to be a significant prognostic moderator, this finding should be validated by conducting further studies using larger cohorts of patients. Another limitation is the overall outline of ITPN molecular alterations and the lack of similar sequencing panels among studies, with no data from whole‐genome and RNA transcriptome sequencing. Lastly, most case reports did not specifically report the use of exclusionary ancillary stains (e.g. acinar stains to exclude the differential diagnosis of an acinar neoplasm), which is an important step to support the diagnosis of this type of lesion. Despite these limitations, the overall findings of this study depict a clear representation of pancreatic ITPN, highlighting their peculiarities in the pathological landscape of pancreatic neoplasms.

In conclusion, this study provides a general description of the most important features of pancreatic ITPNs. They are a distinctive type of pancreatic neoplasm with specific histological, immunohistochemical, and molecular profiles. As highlighted in this study, their recognition during routine diagnostic activity is important, starting from their clinical prognostic implications. In the context of precision oncology and for selected cases, the molecular profile of ITPN should be investigated using specific NGS‐based approaches to identify the potential targets for tailored therapies.

## Author Contributions

CL: study conception and design; GP, CL: systematic review; NV, CL: statistical analysis; all authors: data elaboration, discussion, and interpretation; GP, VNA, CL: paper writing; all authors: final editing and approval of the present version.

## Funding Information

This study was supported by Fondazione Cariverona Oncology Biobank Project “Antonio Schiavi” (prot. 203885/2017); Fondazione Italiana Malattie Pancreas (FIMP‐ J38D19000690001); Italian Ministry of Health (RF CO‐2019‐12369662: CUP: B39C21000370001); Associazione Italiana Ricerca sul Cancro (AIRC IG n. 26343).

## Conflict of Interest

There are no conflicts of interest to report.

## Ethical Statement

Not applicable (review and elaboration of already published data).

## Supporting information


**Figure S1.** PRISMA checklist for this study.

**Figure S2.** Interactive plot of pathway analysis of all cases included in this study. Darker and bigger nodes represent more significantly enriched/larger gene sets included in the pathway. Here, PI3K‐Akt is shown as the most activated pathway in pancreatic ITPN. The statistical associations taking into account all cancer types, based on the pathway analysis, were more significant with breast and hepatocellular cancers rather than with conventional pancreatic cancer (red box).

**Table S1.** Summarizing study‐by‐study table of clinicopathological features of all reported cases of ITPN.

**Table S2.** Summarizing study‐by‐study table of mucins expression in all reported ITPN investigated with immunohistochemistry.

**Table S3.** Summarizing study‐by‐study table of the molecular findings derived from all studies on the genetic profiles of pancreatic ITPN.

## Data Availability

All data/information are available in the article and in the supplementary material.
