# Supplementary material for: Intraductal tubulopapillary neoplasm (ITPN) of the pancreas: a distinct entity among pancreatic tumors
Source: Histopathology. 2022 May 27;81(3):297–309. doi: 10.1111/his.14698 (PMC9544156; doi:10.1111/his.14698)
Supplement: Supplementary file 1 — Figure S1. PRISMA checklist for this study. [file HIS-81-297-s005.docx]

**Supplementary Figure 1.** PRISMA checklist for this study

Records after duplicates removed
(n = 213)

Additional records identified through other sources
(n = 1)

Records identified through database searching
(n = 212)

## Identification

## Screening

Records excluded
(n =74)

Records screened
(n = 213)

Full-text articles excluded, with reasons
(n = 71)

*Doubled cohort (n=3)*

*Other tumor types, including biliary ITPN (n=58)*

*Reviews (n=9)*

*In vitro study (n=1)*

## Eligibility

Full-text articles assessed for eligibility
(n = 139)

Studies included in the systematic review: n=68

## Included
