# Supplementary figures and images for: Intraductal tubulopapillary neoplasm (ITPN) of the pancreas: a distinct entity among pancreatic tumors
Source: Histopathology. 2022 May 27;81(3):297–309. doi: 10.1111/his.14698 (PMC9544156; doi:10.1111/his.14698)

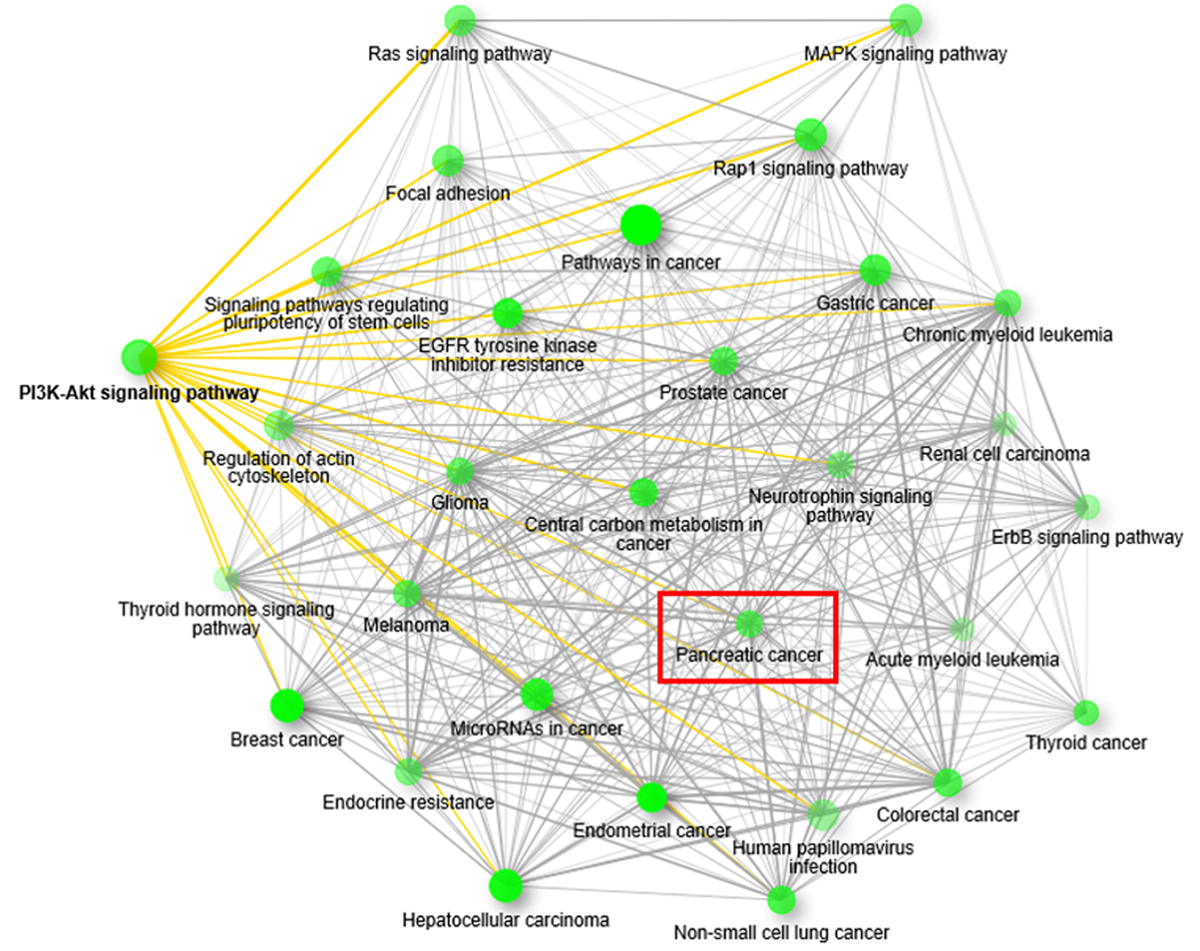

Supplement: Supplementary file 2 — Figure S2. Interactive plot of pathway analysis of all cases included in this study. Darker and bigger nodes represent more significantly enriched/larger gene sets included in the pathway. Here, PI3K‐Akt is shown as the most activated pathway in pancreatic ITPN. The statistical associations taking into account all cancer types, based on the pathway analysis, were more significant with breast and hepatocellular cancers rather than with conventional pancreatic cancer (red box). [file HIS-81-297-s003.tif]
