# Supplementary material for: Intraductal tubulopapillary neoplasm (ITPN) of the pancreas: a distinct entity among pancreatic tumors
Source: Histopathology. 2022 May 27;81(3):297–309. doi: 10.1111/his.14698 (PMC9544156; doi:10.1111/his.14698)
Supplement: Supplementary file 3 — Table S1. Summarizing study‐by‐study table of clinicopathological features of all reported cases of ITPN. [file HIS-81-297-s004.docx]

**Supplementary Table 1**. Summarizing study-by-study table of clinico-pathological features of all reported cases of ITPN

| **Author, year (and study type)** | **Country** | **Sex, age** | **Site in the pancreas** | **Associated cancer** | **Total Size** | **pTNM** | **VI** | **NI** | **Resection status (R)** | **Involved duct(s)** | **Symptom** | **Type of specimen** | **Main radiologic findings** | **Survival** |
| --- | --- | --- | --- | --- | --- | --- | --- | --- | --- | --- | --- | --- | --- | --- |
| Suda et al 1996 (Re) | Japan | M, 55 | H | yes | 70 mm | TxN1M0 | n/a | n/a | n/a | M | n/a | SS | n/a | AF 844 months |
| Suda et al 1996 (Re) | Japan | M, 47 | H | yes | 30 mm | TxN1M0 | n/a | n/a | n/a | M | n/a | SS | n/a | DD 60 months |
| Suda et al 1996 (Re) | Japan | F, 75 | H | yes | 40 mm | TxN1M0 | n/a | n/a | n/a | M | n/a | SS | n/a | AF 48 months |
| Suda et al 1996 (Re) | Japan | F, 78 | H | yes | 30 mm | TxN0M0 | n/a | n/a | n/a | M | n/a | AS | n/a | Diagnosis at autopsy |
| Bakotic et al 1999 (CR) | USA | M, 69 | B-T | no | 9 mm | n/a | n/a | n/a | R0 | M | chronic  pancreatitis | SS | S | n/a |
| Kato et al 2001 (CR) | Japan | M, 70 | B | no | 6 mm | TisN0M0 | no | no | n/a | B | abdominal pain,  diarrhoea | SS | C | n/a |
| Amaris et al 2002 (CR) | France | M, 73 | B-T | no | n/a | TisN0M0 | no | no | n/a | BR | abdominal pain,  dyspepsia | SS | C | n/a |
| Tajiri et al 2004 (Re) | Japan | M, 65 | H | n/a | 55 mm | n/a | n/a | n/a | n/a | M | jaundice, fever | SS | n/a | AF 18 months |
| Tajiri et al 2004 (Re) | Japan | F, 36 | B | n/a | 20 mm | n/a | n/a | n/a | n/a | M | lumbar pain | SS | n/a | AF 48 months |
| Tajiri et al 2004 (Re) | Japan | F, 48 | H | n/a | 26 mm | n/a | n/a | n/a | n/a | M | abdominal pain | SS | n/a | AF 72 months |
| Tajiri et al 2004 (Re) | Japan | M, 67 | T | n/a | 30 mm | n/a | n/a | n/a | n/a | M | lumbar pain | SS | n/a | AF 24 months |
| Ito et al 2005 (CR) | Japan | M, 51 | H | no | 55 mm | TisN0M0 | no | no | R0 | BR | none | EUS-FNAB + SS | S | AF 17 months |
| Itatsu et al 2006 (CR) | Japan | F, 50 | H | no | 8 mm | TisN0M0 | no | no | R0 | M | diarrhoea and abdominal  pain | SS | S | AF 23 months |
| Thirot-Bidault 2006 (CR) | France | M, 67 | T | yes | 30 mm | T1N1M0 | n/a | n/a | n/a | MX | weight loss | SS | SC | n/a |
| Hisa et al 2007 (CR) | Japan | M, 84 | B | no | 28 mm | TisN0M0 | no | no | R0 | M | none | SS | S | DO 15 months |
| Oh et al 2008 (CR) | Korea | F, 63 | B | yes | 25 mm | T1N0M0 | n/a | n/a | n/a | M | abdominal pain, diarrhoea | SS | S | n/a |
| Terada et al 2008 (CR) | Japan | M, 67 | H | yes | n/a | T1N0M0 | no | no | R0 | M | abdominal pain | EUS-FNAB + SS | C | AF 60 months |
| Yamaguchi§ et al 2011 (Re) | Japan | F, 60 | H | no | 60 mm | TisN0M0 | no | no | n/a | n/a | none | SS | n/a | DO 19 months |
| Yamaguchi et al 2011 (Re) | Japan | F, 35 | B | no | 10 mm | TisN0M0 | no | no | n/a | n/a | abdominal pain | SS | n/a | AF 72 months |
| Yamaguchi et al 2011 (Re) | Japan | F, 68 | H | no | 25 mm | TisN0M0 | no | no | n/a | n/a | none | SS | n/a | AF 29 months |
| Yamaguchi et al 2011 (Re) | Japan | M, 53 | B | no | 20 mm | TisN0M0 | no | no | n/a | M | abdominal pain | SS | n/a | AF 36 months |
| Yamaguchi et al 2011 (Re) | Japan | F, 60 | H | no | 45 mm | TisN0M0 | no | no | n/a | n/a | abdominal pain | SS | n/a | AF 24 months |
| Yamaguchi et al 2011 (Re) | Japan | F, 73 | H | no | 52 mm | TisN0M0 | no | no | n/a | n/a | none | SS | n/a | AF 33 months |
| Yamaguchi et al 2011 | Japan | M, 72 | B | yes | 10 mm | TxN0M0 | yes | n/a | n/a | n/a | none | SS | n/a | AF 33 months |
| Yamaguchi et al 2011 (Re) | Japan | M, 44 | H | yes | 60 mm | TxN0M0 | yes | n/a | n/a | n/a | abdominal pain | SS | n/a | AF 72 months |
| Yamaguchi et al 2011 (Re) | Japan | M, 48 | diffuse | yes | 150 mm | TxN0M0 | yes | n/a | n/a | n/a | jaundice | SS | n/a | DD 7 months |
| Yamaguchi et al 2011 (Re) | Japan | M, 70 | diffuse | no | 40 mm | TisN0M0 | no | no | n/a | n/a | exacerbation of DM | SS | n/a | AF 24 months |
| Yamaguchi et al 2011 (Re) | Japan | F, 62 | n/a | yes | n/a | n/a | n/a | n/a | n/a | n/a | none | SS | n/a | AF 7 months |
| Yamaguchi et al 2011 (Re) | Japan | M, 68 | n/a | no | n/a | n/a | n/a | n/a | n/a | n/a | none | SS | n/a | AF 2 months |
| Shimizu et al 2011 (CR) | Japan | M, 63 | T | yes | 10 mm | T1N0M0 | yes | no | n/a | M | acute pancreatitis | SS | C | AF 72 months |
| Bhuva et al 2011 (CR) | UK | M, 50 | H | yes | n/a | T2N1M0 | yes | n/a | n/a | M | abdominal pain, jaundice, anaemia | EUS-FNAB + SS | SC | AD 28 months |
| Jokoji R et al 2012 (CR) | Japan | M, 68 | B | yes | 18 mm | TxN0M0 | n/a | n/a | n/a | M | abdominal pain | BC + SS | S | AF 15 months |
| Urata T et al 2012 (CR) | Japan | F, 78 | B | yes | n/a | TxN0M0 | n/a | n/a | R0 | M | none | EUS-FNAB + SS | S | AD 43 months |
| Tajiri T et al 2012 (CR) | Japan | M, 66 | H | no | n/a | TisN0M0 | no | no | n/a | n/a | appetite loss | SS | SC | AF 12 months |
| Shibasaki Y et al 2012 (CR) | Japan | M, 61 | diffuse | yes | 115 mm | T3N0M0 | yes | no | R1 | MX | exacerbation of DM | SS | S | AF 14 months |
| Guan H et al 2012 (CR) | USA | F, 41 | H | no | 23 mm | TisN0M0 | no | no | n/a | BR | none | EUS-FNAC + SS | SC | n/a |
| Motosugi U et al 2012 (Re) | Japan | 3M, 2F | 2 H, 2 B, 1 H-B | n/a | 6 - 81 mm | n/a | n/a | n/a | n/a | M | n/a | SS | C | n/a |
| Kasugai H et al 2013 (CR) | Japan | F, 69 | diffuse | no | 120 mm | TisN0M0 | no | no | n/a | M | excessive thirst | BC + SS | S | AF 24 months |
| Furuhata A et al, 2013 (CR) | Japan | M, 74 | H | yes | 70 mm | TxN0M0 | n/a | n/a | n/a | n/a | Fever | EUS-FNAC + SS | S | n/a |
| Matsushita K et al, 2013 (CR) | Japan | F, 47 | H | yes | n/a | n/a | n/a | n/a | R0 | M | none | SS | S | AF 30 months |
| Chang X et al, 2014 (Re) | China | 2M, 4F; 48-70 | 4 H, 1 B, 1 T | n/a | 15 - 45 mm | n/a | n/a | n/a | n/a | BR | 4 abdominal pain, 1 jaundice, 1 none | SS | n/a | n/a |
| Someya Y et al, 2014 (CR) | Japan | M, 74 | H | yes | 70 mm | n/a | n/a | n/a | n/a | n/a | Fever | SS | S | AF 24 months |
| Matsuda M et al, 2014 (CR) | Japan | M, 71 | B | yes | 90 mm | n/a | yes | n/a | n/a | M | none | SS | S | DD 21.5 months |
| Del Chiaro et al, 2014 (CR) | Italy | M, 78 | H | no | 11 mm | TisN0M0 | no | no | n/a | MX | abdominal pain | SS | S | n/a |
| Ahls MG et al, 2014 (CR) | Germany | F, 43 | H | no | 26 mm | TisN0M0 | no | no | R0 | M | epigastric pain | SS | S | n/a |
| Ito H et al, 2014 (CR) | Japan | M, 75 | B | yes | n/a | n/a | no | n/a | R0 | M | none | SS | S | n/a |
| Takayama S, 2015 (CR) | Japan | F, 54 | H | yes | 50 mm | T3N0M0 | n/a | n/a | n/a | MX | diarrhoea | EUS-FNAB + SS | S | AF 10 months |
| Yoshida Y et al, 2015 (CR) | Japan | M, 75 | H | no | 12 mm | TisN0M0 | no | no | R0 | BR | none | EUS-FNAB + SS | S | n/a |
| Kitaguchi K et al, 2015 (CR) | Japan | M, 61 | H | yes | 12 mm | TxN0M0 | no | no | R0 | M | none | SS | S | AF 22 months |
| Matthews Y et al, 2015 (CR) | Australia | M, 55 | T | yes | 100 mm | T3N1M1 | n/a | n/a | n/a | n/a | abdominal pain | SS | C | AD 36 months |
| Kolby et al, 2015 (CR) | Sweden | M, 42 | diffuse | yes | 35 mm | T3N0M0 | n/a | n/a | R0 | M | abdominal pain | SS | S | AF 19 months |
| Tajima, 2015 (CR) | Japan | M, 80 | H-B | no | 5 mm | n/a | no | no | R0 | M | none | EUS-FNAC + SS | S | AF 12 months |
| Savant et al, 2016 (CR) | USA | F, 82 | B | no | 40 mm | n/a | no | no | n/a | M | appetite loss | EUS-FNAC + SS | S | n/a |
| Date et al, 2016 (Re) | Japan | F, 54 | H | yes | 55 mm | T2N0M0 | n/a | n/a | R0 | M | abdominal pain, anorexia | SS | SC | AF 24 months |
| Niu et al, 2017 (CR) | China | M, 38 | H | yes | 42 mm | n/a | yes | no | no | n/a | mild jaundice | SS | S | n/a |
| Fujimoto et al 2017 (CR) | Japan | M, 74 | H-B | yes | n/a | TxN2Mx | no | n/a | n/a | M | none | SS | SC | AF 9 months |
| Kovacevic et al 2017 (CR) | Denmark | M, 68 | H | n/a | n/a | n/a | n/a | n/a | n/a | M | abdominal pain | EUS-FNAB | C | AF 6 months |
| Basturk et al, 2017 (Re) | n/a | F, 51 | H | yes | 25 mm | n/a | n/a | n/a | n/a | n/a | abdominal pain | SS | n/a | DD 41 months |
| Basturk et al, 2017 (Re) | n/a | F, 63 | diffuse | yes | 45 mm | n/a | n/a | n/a | n/a | n/a | abdominal pain, steatorrhea | SS | n/a | DOC 49 months |
| Basturk et al, 2017 (Re) | n/a | F, 53 | H | yes | 15 mm | n/a | n/a | n/a | n/a | n/a | abdominal pain, discomfort | SS | n/a | DD 23 months |
| Basturk et al, 2017 (Re) | n/a | F, 36 | diffuse | yes | n/a | n/a | n/a | n/a | n/a | n/a | none | SS | n/a | AD 123 months |
| Basturk et al, 2017 (Re) | n/a | F, 65 | diffuse | no | n/a | n/a | n/a | n/a | n/a | n/a | abdominal pain | SS | n/a | AD 16 months |
| Basturk et al, 2017 (Re) | n/a | F, 25 | T | yes | 100 mm | n/a | n/a | n/a | n/a | n/a | none | SS | n/a | AF 18 months |
| Basturk et al, 2017 | n/a | M, 61 | H | yes | 80 mm | n/a | n/a | n/a | n/a | n/a | abdominal pain, weight loss | SS | n/a | AD 12 months |
| Basturk et al, 2017 (Re) | n/a | F, 58 | T | yes | 90 mm | n/a | n/a | n/a | n/a | n/a | none | SS | n/a | AD 173 months |
| Basturk et al, 2017 (Re) | n/a | F, 72 | H | yes | 60 mm | n/a | n/a | n/a | n/a | n/a | none | SS | n/a | AD 87 months |
| Basturk et al, 2017 (Re) | n/a | M, 38 | n/a | n/a | 40 mm | n/a | n/a | n/a | n/a | n/a | none | SS | n/a | AD 51 months |
| Basturk et al, 2017 (Re) | n/a | M, 53 | H | yes | 54 mm | n/a | n/a | n/a | n/a | n/a | abdominal pain | SS | n/a | AD 164 months |
| Basturk et al, 2017 (Re) | n/a | M, 45 | diffuse | yes | 5 mm | n/a | n/a | n/a | n/a | n/a | abdominal pain | SS | n/a | n/a |
| Basturk et al, 2017 (Re) | n/a | M, 60 | n/a | yes | n/a | n/a | n/a | n/a | n/a | n/a | none | SS | n/a | n/a |
| Basturk et al, 2017 (Re) | n/a | M, 49 | n/a | yes | n/a | n/a | n/a | n/a | n/a | n/a | none | SS | n/a | n/a |
| Basturk et al, 2017 (Re) | n/a | F, 53 | H | yes | 50 mm | n/a | n/a | n/a | n/a | n/a | none | SS | n/a | n/a |
| Basturk et al, 2017 (Re) | n/a | F, 62 | diffuse | yes | n/a | n/a | n/a | n/a | n/a | n/a | none | SS | n/a | n/a |
| Basturk et al, 2017 (Re) | n/a | n/a | n/a | no | n/a | n/a | n/a | n/a | n/a | n/a | none | SS | n/a | n/a |
| Basturk et al, 2017 (Re) | n/a | F, 56 | B | no | 25 mm | n/a | n/a | n/a | n/a | n/a | abdominal pain | SS | n/a | AD 72 months |
| Basturk et al, 2017 (Re) | n/a | M, 36 | n/a | no | n/a | n/a | n/a | n/a | n/a | n/a | none | SS | n/a | AF 1 months |
| Basturk et al, 2017 (Re) | n/a | F, 71 | T | no | 50 mm | n/a | n/a | n/a | n/a | n/a | none | SS | n/a | AD 120 months |
| Basturk et al, 2017 (Re) | n/a | M, 53 | T | yes | 20 mm | n/a | n/a | n/a | n/a | n/a | none | SS | n/a | AF 95 months |
| Basturk et al, 2017 (Re) | n/a | M, 53 | n/a | n/a | n/a | n/a | n/a | n/a | n/a | n/a | abdominal pain | SS | n/a | n/a |
| Basturk et al, 2017 (Re) | n/a | M, 50 | n/a | yes | n/a | n/a | n/a | n/a | n/a | n/a | abdominal pain | SS | n/a | AF 77 months |
| Basturk et al, 2017 (Re) | n/a | F, 73 | T | yes | 37 mm | n/a | n/a | n/a | n/a | n/a | abdominal pain, nausea, vomiting | SS | n/a | AF 64 months |
| Basturk et al, 2017 (Re) | n/a | F, 79 | H | yes | 90 mm | n/a | n/a | n/a | n/a | n/a | abdominal pain, weight loss | SS | n/a | AD 37 months |
| Basturk et al, 2017 (Re) | n/a | M, 75 | T | no | 15 mm | n/a | n/a | n/a | n/a | n/a | n/a | SS | n/a | AD 20 months |
| Basturk et al, 2017 (Re) | n/a | M, 64 | H | yes | 30 mm | n/a | n/a | n/a | n/a | n/a | epigastric pain | SS | n/a | AF 19 months |
| Basturk et al, 2017 (Re) | n/a | M, 67 | H | yes | 35 mm | n/a | n/a | n/a | n/a | n/a | abdominal pain | SS | n/a | AF 13 months |
| Basturk et al, 2017 (Re) | n/a | F, 40 | H | no | 30 mm | n/a | n/a | n/a | n/a | n/a | none | SS | n/a | n/a |
| Basturk et al, 2017 (Re) | n/a | F, 57 | n/a | no | 10 mm | n/a | n/a | n/a | n/a | n/a | none | SS | n/a | AD 16 months |
| Basturk et al, 2017 (Re) | n/a | n/a | n/a | no | n/a | n/a | n/a | n/a | n/a | n/a | none | SS | n/a | n/a |
| Basturk et al, 2017 (Re) | n/a | F, 53 | n/a | yes | 60 mm | n/a | n/a | n/a | n/a | n/a | none | SS | n/a | n/a |
| Basturk et al, 2017 (Re) | n/a | F, 46 | n/a | yes | 100 mm | n/a | n/a | n/a | n/a | n/a | none | SS | n/a | n/a |
| Kuscher et al, 2017 (CR) | Austria | M, 73 | H | yes | 28 mm | T2N0M0 | n/a | n/a | R0 | M | wright loss | BC + SS | C | AF 6 months |
| Maghrebi et al, 2017 (CR) | Tunisia | M, 62 | H | n/a | n/a | n/a | n/a | n/a | n/a | M | abdominal pain, nausea, vomiting | SS | C | AF 12 months |
| Umemura et al, 2017 (CR) | Japan | F, 50 | B | n/a | 15 mm | n/a | no | no | R0 | MX | left flank pain and vomiting | SS | S | Recurrence after 36 months |
| Umemura et al, 2019* (CR) | Japan | same patient, 53 (upper described) | H | no | 20 mm | n/a | no | no | R0 | M | none (diagnosis on follow-up) | SS | S | AF 48 months |
| Inomata et al, 2018 (CR) | Japan | F, 55 | H | no | n/a | TisN0M0 | no | no | n/a | n/a | abdominal pain, anaemia | SS | SC | AF 34 months |
| Sakamoto et al, 2018 (CR) | Japan | M, 72 | H | n/a | n/a | n/a | n/a | n/a | n/a | M | abdominal tenderness and epigastric rigidity | BC + SS | SC | AF 12 months |
| Saeki et al, 2018 (CR) | Japan | M, 54 | H | no | 20 mm | n/a | no | no | R0 | M | none | BC + SS | S | Recurrence after 192 months |
| Saeki et al, 2018* (CR) | Japan | same patient, 70 (upper described) | B | yes | 5 mm | n/a | n/a | n/a | n/a | M | none (diagnosis on follow-up) | SS | S | AF 9 months |
| Cauthen et al, 2018 (CR) | USA | F, 74 | H | no | 55 mm | TisN0M0 | no | no | R0 | M | abdominal pain, weight loss | SS | SC | n/a |
| Zhang et al, 2019 (CR) | China | F, 36 | H | yes | 40 mm | T2N1cM0 | yes | n/a | R1 | M | jaundice, diarrhoea | EUS-FNAB + SS | S | AD 2 months |
| Zhang et al, 2019 (CR) | China | F, 62 | H | yes | 32 mm | T2N0Mx | n/a | n/a | n/a | M | n/a | SS | S | n/a |
| Fritz et al, 2019 (CR) | Germany | M, 68 | H | yes | 59 mm | T3N0M0 | n/a | n/a | R0 | n/a | jaundice, weight loss | SS | S | AF 6 months |
| Ko et al, 2019 (CR) | Japan | M, 61 | H | yes | 10 mm | n/a | n/a | n/a | R0 | M | none | BC + SS | S | Recurrence after 16 months |
| Ko et al, 2019* (CR) | Japan | same patient, 62 (upper described) | B | no | 20 mm | n/a | n/a | n/a | n/a | M | none (diagnosis on follow-up) | SS | S | AF 23 months |
| Kim et al, 2019 (Re) | South Korea | 5 M, 2 F; 34-71 | 3 H, 2 B, 1 H-B, 2 B-T | 5 yes, 3 no | 12.7 - 98 mm | n/a | n/a | n/a | n/a | 7 M, 1 BR | n/a | SS | S | n/a |
| Dalal et al, 2019 (CR) | India | M, 53 | H | n/a | 16 mm | n/a | n/a | n/a | n/a | M | epigastric pain and weight loss | Surgical biopsy + SS | S | n/a |
| Zhou et al, 2019 (CR) | China | F, 35 | T | yes | 90 mm | n/a | n/a | n/a | n/a | M | none (follow-up of a known lesion) | SS | SC | AF 12 months |
| Huang et al, 2020 (CR) | USA | F, 72 | B-T | n/a | 12 mm | n/a | n/a | n/a | n/a | n/a | none | EUS-FNAB | SC | n/a |
| Kosmidis et al, 2020 (CR) | Greece | M, 82 | H-B | yes | 85 and 45 mm | T3N0M0 | n/a | yes | n/a | MX | abdominal pain, weight loss, anorexia | CT-guided biopsy + SS | C | AD 28 months |
| Nabrinsky et al , 2020 (CR) | USA | F, 52 | H | yes | 135 mm | T3N0M0 | n/a | n/a | R0 | BR | abdominal pain, weight loss | SS | S | n/a |
| Zhong-fei et al, 2020 (CR) | China | F, 63 | B-T | n/a | n/a | n/a | n/a | n/a | biopsy only | M | Nausea and upper abdominal pain | EUS-FNAB | S | DD 9 months |
| Shimizu et al, 2020 (CR) | Japan | F, 73 | B | yes | 37 mm | T2N0M0 | n/a | n/a | R0 | M | epigastric discomfort and pain | SS | S | n/a |
| Paik, 2020 (CR) | South Korea | M, 64 | diffuse | yes | n/a | n/a | n/a | n/a | n/a | M | abdominal pain, weight loss | SS | S | DOC 48 months |
| Liu et al, 2020 (CR) | China | M, 60 | diffuse | n/a | n/a | n/a | n/a | n/a | n/a | MX | recurrent pancreatitis | SS | C | n/a |
| Cohen et al, 2020 (CR) | USA | M, 68 | B-T | yes | 115 mm | T3N0M0 | n/a | n/a | R0 | MX | none (follow-up after pancreaticoduodenectomy) | EUS-FNAB + SS | SC | AF 8 months |
| Yamaguchi et al, 2021 (CR) | Japan | M, 85 | H | yes | 25 mm | T1N0M0 | no | n/a | R0 | BR | none (follow-up after colonic surgery) | BC + SS | SC | AF 48 months |

Abbreviations: Re: retrospective; CR; case report; H, head; B, body; T, tail; n/a, not available; VI: vascular invasion; NI: perineural invasion; M, main duct; BR, branch duct; MX, mixed; DM: diabetes mellitus; SS: surgical specimens; AS: autopsy-derived specimen; EUS-FNAB: ultrasound-guided fine needle aspiration biopsy; EUS-FNAC: ultrasound-guided fine needle aspiration cytology; BC: brushing-cytology; C = radiologic cystic appearance; S: radiologic solid / mass-forming lesion; SC: radiologic mixed cystic- solid appearance; AF, alive free of disease; AD, alive with disease; DOC, dead of other causes; DD, dead with disease. Notes: *patients derived from 3 studies with overlapped cohort [see references 24,25,34].
