# Supplementary material for: Intraductal tubulopapillary neoplasm (ITPN) of the pancreas: a distinct entity among pancreatic tumors
Source: Histopathology. 2022 May 27;81(3):297–309. doi: 10.1111/his.14698 (PMC9544156; doi:10.1111/his.14698)
Supplement: Supplementary file 4 — Table S2. Summarizing study‐by‐study table of mucins expression in all reported ITPN investigated with immunohistochemistry. [file HIS-81-297-s002.docx]

**Supplementary Table 2**: Summarizing study-by-study table of mucins expression in all reported ITPN investigated with immunohistochemistry

| **Author, year** | **MUC1** | **MUC2** | **MUC5AC** | **MUC6** |
| --- | --- | --- | --- | --- |
| Tajiri, 2004 | +^1^ | -^6^ | -^11^ | NA |
| Tajiri, 2004 | +^1^ | -^6^ | -^11^ | NA |
| Tajiri, 2004 | +^1^ | -^6^ | -^11^ | NA |
| Tajiri, 2004 | -^1^ | -^6^ | -^11^ | NA |
| Itatsu, 2006 | +^0^ | - ^0^ | -^0^ | NA |
| Thirot-Bidault, 2006 | +^0^ | - ^0^ | -^0^ | NA |
| Hisa, 2007 | focally +^1^ | -^6^ | -^11^ | focally +^14^ |
| Oh, 2008 | +^0^ | -^0^ | -^0^ | NA |
| Terada, 2008 | -^1^ | +^6^ | +^11^ | +^14^ |
| Yamaguchi, 2011 | +^1^ | -^6^ | -^11^ | -^15^ |
| Yamaguchi, 2011 | +^1^ | -^6^ | -^11^ | +^15^ |
| Yamaguchi, 2011 | +^1^ | -^6^ | -^11^ | +^15^ |
| Yamaguchi, 2011 | +^1^ | -^6^ | -^11^ | -^15^ |
| Yamaguchi, 2011 | +^1^ | -^6^ | -^11^ | -^15^ |
| Yamaguchi, 2011 | +^1^ | -^6^ | -^11^ | +^15^ |
| Yamaguchi, 2011 | +^1^ | -^6^ | -^11^ | +^15^ |
| Yamaguchi, 2011 | +^1^ | -^6^ | -^11^ | +^15^ |
| Yamaguchi, 2011 | +^1^ | -^6^ | -^11^ | -^15^ |
| Yamaguchi, 2011 | +^1^ | -^6^ | -^11^ | +^15^ |
| Bhuva, 2011 | NA | -^0^ | -^0^ | +^0^ |
| Jokoji, 2012 | focally +^1^ | -^6^ | -^11^ | -^14^ |
| Urata, 2012 | +^0^ | -^0^ | -^0^ | -^0^ |
| Tajiri, 2012 | +^1^ | -^6^ | -^11^ | +^14^ |
| Shibasaki, 2012 | +^0^ | -^0^ | -^0^ | -^0^ |
| Guan, 2012 | +^0^ | -^0^ | NA | NA |
| Kasugai, 2013 | +^0^ | NA | -^0^ | +^0^ |
| Furuhata, 2013 | +^0^ | -^0^ | -^0^ | +^0^ |
| Matsushita, 2013 | +^0^ | -^0^ | -^0^ | NA |
| Chang, 2014 | 3/6 +^1^ | 6/6 -^6^ | 3/6 +^11^ | NA |
| Someya, 2014 | +^0^ | -^0^ | -^0^ | +^0^ |
| Matsuda, 2014 | +^0^ | -^0^ | -^0^ | focally +^0^ |
| Del Chiaro, 2014 | +^0^ | -^0^ | -^0^ | +^0^ |
| Ahls, 2014 | +^0^ | -^0^ | -^0^ | +^0^ |
| Ito, 2014 | +^0^ | -^0^ | -^0^ | NA |
| Takayama, 2015 | +^2^ | -^6^ | -^11^ | NA |
| Yoshida, 2015 | +^0^ | NA | -^0^ | +^0^ |
| Matthews, 2015 | +^0^ | -^0^ | -^0^ | NA |
| Tajima, 2015 | +^0^ | -^0^ | -^0^ | +^0^ |
| Date, 2016 | +^0^ | -^0^ | -^0^ | +^0^ |
| Niu, 2017 | +^3^ | -^7^ | NA | NA |
| Fujimoto, 2017 | +^4^ | -^8^ | -^12^ | -^16^ |
| Kovacevic, 2017 | +^0^ | NA | focally +^0^ | focally +^0^ |
| Basturk, 2017 | 15/17 +^5^ | 17/17 -^9^ | 1/24 +^12^ | 17/25 +^17^ |
| Kuscher, 2017 | NA | -^10^ | -^13^ | NA |
| Umemura, 2017 | +^0^ | -^0^ | -^0^ | -^0^ |
| Umemura, 2019* | +^0^ | -^0^ | -^0^ | focally +^0^ |
| Inomata, 2018 | +^0^ | -^0^ | -^0^ | +^0^ |
| Sakamoto, 2018 | +^0^ | -^0^ | NA | NA |
| Saeki, 2018 | focally +^0^ | -^0^ | -^0^ | +^0^ |
| Saeki, 2018* | focally +^0^ | -^0^ | -^0^ | +^0^ |
| Ko, 2019 | NA | -^0^ | -^0^ | +^0^ |
| Ko, 2019* | NA | -^0^ | focally +^0^ | -^0^ |
| Zhou, 2019 | NA | -^0^ | -^0^ | NA |
| Kosmidis, 2020 | +^0^ | -^0^ | NA | NA |
| Shimizu, 2020 | +^0^ | -^0^ | -^0^ | +^0^ |
| Liu, 2020 | +^0^ | -^0^ | NA | +^0^ |
| Yamaguchi, 2021 | +^0^ | -^0^ | -^0^ | -^0^ |

Abbreviations: ITPN, intraductal tubulopapillary neoplasm; NA, not available. Notes: ^for calculating the overall results, the percentage was calculated on the total of those cases with a clearly reported expression for the specific marker; cases without IHC data are indicated as NA; *cases from the same patient listed in the previous row.

Notes: Type of antibody, clone and company used in the study: ^0^= unspecified clone and company; ^1^=MUC1 (Ma695, Novocastra); ^2^=MUC1 (Ma552; Novocastra); ^3^=MUC1 (ZM‑0391, EnVision Immunity); ^4^=MUC1 (unspecified clone, Leica Microsystems); ^5^=MUC1 (unspecified clone, Vector Laboratories); ^6^=MUC2 (Ccp-8, Novocastra); ^7^=MUC2 (ZM‑0392, EnVision Immunity); ^8^=MUC2 (unspecified clone, Leica Microsystems); ^9^=MUC2 (unspecified clone, Vector Laboratories); ^10^=MUC2 (IR658, Dako); ^11^=MUC5AC (CLH2; Novocastra); ^12^=MUC5AC (unspecified clone, Leica Microsystems); ^13^=MUC5AC (IR661, Dako); ^14^= MUC6 (CLH5; Novocastra); ^15^= MUC6 /CLH5, Vector Laboratories); ^16^= MUC6 (unspecified clone, Leica Microsystems); ^17^= MUC6 (unspecified clone, B.D. Pharmingen).
