# Supplementary material for: Intraductal tubulopapillary neoplasm (ITPN) of the pancreas: a distinct entity among pancreatic tumors
Source: Histopathology. 2022 May 27;81(3):297–309. doi: 10.1111/his.14698 (PMC9544156; doi:10.1111/his.14698)
Supplement: Supplementary file 5 — Table S3. Summarizing study‐by‐study table of the molecular findings derived from all studies on the genetic profiles of pancreatic ITPN. [file HIS-81-297-s001.docx]

**Supplementary Table 3**: Summarizing study-by-study table of the molecular findings derived from all studies on the genetic profiles of pancreatic ITPN.

| **Author, year** | **Altered gene** | **Type of alteration** | **Consequence** | **Type of molecular analysis** | **Type of specimen** | **Type of tissue** |
| --- | --- | --- | --- | --- | --- | --- |
| Bakotic, 1999 | *KRAS* | Missense mutation G12R | Non-synonymous coding | Sanger sequencing | Surgical specimen | NA |
| Kato, 2001 | *KRAS* | Somatic mutation G12X | Non-synonymous coding | Sanger sequencing | Surgical specimen | FFPE-(DS) |
| Yamaguchi, 2011 | none detected | - | - | Sanger sequencing | Surgical specimen | FFPE-(DS) |
| Yamaguchi, 2011 | none detected | - | - | Sanger sequencing | Surgical specimen | FFPE-(DS) |
| Yamaguchi, 2011 | none detected | - | - | Sanger sequencing | Surgical specimen | FFPE-(DS) |
| Yamaguchi, 2011 | none detected | - | - | Sanger sequencing | Surgical specimen | FFPE-(DS) |
| Yamaguchi, 2011 | *PIK3CA* | Missense mutation G1049R | Non-synonymous coding | Sanger sequencing | Surgical specimen | FFPE-(DS) |
| Yamaguchi, 2011 | none detected | - | - | Sanger sequencing | Surgical specimen | FFPE-(DS) |
| Yamaguchi, 2011 | *PIK3CA* | Missense mutation H1047R | Non-synonymous coding | Sanger sequencing | Surgical specimen | FFPE-(DS) |
| Yamaguchi, 2011 | none detected | - | - | Sanger sequencing | Surgical specimen | FFPE-(DS) |
| Yamaguchi, 2011 | none detected | - | - | Sanger sequencing | Surgical specimen | FFPE-(DS) |
| Yamaguchi, 2011 | *PIK3CA* | Missense mutation E545K | Non-synonymous coding | Sanger sequencing | Surgical specimen | FFPE-(DS) |
| Yamaguchi, 2011 | none detected | - | - | Sanger sequencing | Surgical specimen | FFPE-(DS) |
| Urata, 2012 | *BRAF* | Missense mutation V600E | Non-synonymous coding | Whole-exome sequencing | Surgical specimen | FFPE-(NOS) |
| Tajiri, 2012 | *BRAF* | Missense mutation V600E | Non-synonymous coding | Sanger sequencing | Surgical specimen | FFPE-(DS) |
| Shibasaki, 2012 | none detected (tested gene: *KRAS*) | - | - | NA | NA | NA |
| Kasugai, 2013 | none detected | - | - | NA | NA | NA |
| Chang, 2014 | *KRAS* | Missense mutation G12D | Non-synonymous coding | Sanger sequencing | Surgical specimen | FFPE-(MDS) |
| Chang, 2014 | *KRAS* | Missense mutation G12V | Non-synonymous coding | Sanger sequencing | Surgical specimen | FFPE-(MDS) |
| Ahls MG, 2014 | none detected (tested genes: *BRAF, KRAS, PIK3CA, GNAS, CTNNB1*) | - | - | Sanger sequencing | Surgical specimen | FFPE-(MDS) |
| Kolby, 2015 | none detected (tested genes: *BRAF, KRAS*) | - | - | NA | NA | NA |
| Niu, 2017 | *TP53* | Missense mutation A2030T | Non-synonymous coding | Sanger sequencing | Surgical specimen | FFPE-(NOS) |
| Basturk, 2017 | *ARHGAP26* | Missense mutation R103Q | Non-synonymous coding | Targeted-NGS (MSK-IMPACT) | Surgical specimen | FFPE-(DS) |
|  | *DNMT3A* | Missense mutation R181H | Non-synonymous coding |  |  |  |
|  | *JAK* | Missense mutation V1009D | Non-synonymous coding |  |  |  |
|  | *MCL* | Amplification |  |  |  |  |
|  | *MLL2* | Nonsense mutation E3587* |  |  |  |  |
|  | *ROR2* | Missense mutation R522Q | Non-synonymous coding |  |  |  |
|  | *TET2* | Missense mutation E755K | Non-synonymous coding |  |  |  |
| Basturk, 2017 | *MLL2* | Nonsense mutation R2471* |  | Targeted-NGS (MSK-IMPACT) | Surgical specimen | FFPE-(DS) |
|  | *MLL2* | Frame_Shift_Ins p.L4518fs |  |  |  |  |
| Basturk, 2017 | *BCOR* | Frame_Shift_Ins p.E1484fs |  | Targeted-NGS (MSK-IMPACT) | Surgical specimen | FFPE-(DS) |
|  | *DNMT3B* | Missense mutation G511C | Non-synonymous coding |  |  |  |
|  | *IRF4* | Missense mutation E130G | Non-synonymous coding |  |  |  |
|  | *MCL* | Amplification |  |  |  |  |
|  | *NOTCH2* | Missense mutation N632S | Non-synonymous coding |  |  |  |
| Basturk, 2017 | none detected | - | - | Targeted-NGS (MSK-IMPACT) | Surgical specimen | FFPE-(DS) |
| Basturk, 2017 | *FAM123B* | Splice_Site p.Q2048fs |  | Targeted-NGS (MSK-IMPACT) | Surgical specimen | FFPE-(DS) |
|  | *MAP2K1* | In_Frame_Ins p.59_60insQK |  |  |  |  |
|  | *MAP2K1* | Missense mutation E51G | Non-synonymous coding |  |  |  |
|  | *MCL* | Amplification |  |  |  |  |
|  | *MLL3* | Framseshift_Del |  |  |  |  |
| Basturk, 2017 | *BRCA2* | Missense mutation G1771D | Non-synonymous coding | Targeted-NGS (MSK-IMPACT) | Surgical specimen | FFPE-(DS) |
|  | *EPHA2* | Missense mutation A112T | Non-synonymous coding |  |  |  |
|  | *MCL* | Amplification |  |  |  |  |
| Basturk, 2017 | *CDKN2A* | Loss of expression |  | Targeted-NGS (MSK-IMPACT) and WGS (HiSeqX) | Surgical specimen | FFPE-(DS) (NGS) and frozen tissue (WGS) |
|  | *MLL3* | Missense mutation K992M |  |  |  |  |
|  | *NPM1* | Missense mutation S125L | Non-synonymous coding |  |  |  |
|  | *PTEN* | In_Frame_Del p.T319 | Non-synonymous coding |  |  |  |
| Basturk, 2017 | *MCL* | Amplification |  | Targeted-NGS (MSK-IMPACT) | Surgical specimen | FFPE-(DS) |
| Basturk, 2017 | *BAP1* | Splice_Site p.E577_splice |  | Targeted-NGS (MSK-IMPACT) | Surgical specimen | FFPE-(DS) |
| Basturk, 2017 | *INPP4A* | Missense mutation N308T | Non-synonymous coding | Targeted-NGS (MSK-IMPACT) | Surgical specimen | FFPE-(DS) |
|  | *CDKN2A* | Loss of expression |  |  |  |  |
|  | *KDR* | Missense mutation M559I | Non-synonymous coding |  |  |  |
| Basturk, 2017 | *AXIN1* | Missense mutation E195* | Non-synonymous coding | Targeted-NGS (MSK-IMPACT) and WGS (HiSeqX) | Surgical specimen | FFPE-(DS) (NGS) and frozen tissue (WGS) |
|  | *BAP1* | Missense mutation R213H | Non-synonymous coding |  |  |  |
|  | *FLT4* | Splice_Site p.P1023R |  |  |  |  |
|  | *PBRM1* | Missense mutation | Non-synonymous coding |  |  |  |
|  | *FGFR2* | Fusion gene (partner: *CEP55*) | Protein fusion: in frame (FGFR2-CEP55) |  |  |  |
| Basturk, 2017 | *XPO1* | Missense mutation L660F | Non-synonymous coding | Targeted-NGS (MSK-IMPACT) | Surgical specimen | FFPE-(DS) |
|  | *FGFR2* | Fusion gene (partner: *SASS6*) | Protein fusion: in frame (FGFR2-SASS6) |  |  |  |
| Basturk, 2017 | *EED* | Missense mutation N194S | Non-synonymous coding | Targeted-NGS (MSK-IMPACT) | Surgical specimen | FFPE-(DS) |
|  | *FGFR4* | Framshift p.R464Pfs*32 |  |  |  |  |
|  | *PIK3CA* | Missense mutation G1049R | Non-synonymous coding |  |  |  |
| Basturk, 2017 | *ATRX* | Missense mutation I47V | Non-synonymous coding | Targeted-NGS (MSK-IMPACT) | Surgical specimen | FFPE-(DS) |
|  | *BAP1* | Nonsense mutation S319* |  |  |  |  |
|  | *CRKL* | Missense mutation G136E | Non-synonymous coding |  |  |  |
|  | *MLL1* | Missense mutation I962V | Non-synonymous coding |  |  |  |
|  | *PIK3CA* | Missense mutation H1047R | Non-synonymous coding |  |  |  |
|  | *SPEN* | Missense mutation A3060V | Non-synonymous coding |  |  |  |
|  | *FGFR2* | Fusion gene (partner: *DISP1*) | Protein fusion: out of frame (DISP1-FGFR2) |  |  |  |
|  | *FGFR2* | Fusion gene (partner: *TXLNA*) | Protein fusion: in frame (FGFR2-TXLNA) |  |  |  |
| Basturk, 2017 | *CDKN2A* | Loss of expression |  | Targeted-NGS (MSK-IMPACT) | Surgical specimen | FFPE-(DS) |
|  | *NTRK1* | Missense mutation G181E | Non-synonymous coding |  |  |  |
|  | *SMARCA4* | Missense mutation R885C | Non-synonymous coding |  |  |  |
|  | *FGFR2* | Fusion gene (partner: *VCL*) | Protein fusion: mid-exon (FGFR2-VCL) |  |  |  |
| Basturk, 2017 | *ZFHX3* | Deletion p.G3517_G3527del |  | Targeted-NGS (MSK-IMPACT) | Surgical specimen | FFPE-(DS) |
| Basturk, 2017 | *MCL* | Amplification |  | Targeted-NGS (MSK-IMPACT) and WGS (HiSeqX) | Surgical specimen | FFPE-(DS) (MSK-IMPACT) and frozen tissue (WGS) |
| Basturk, 2017 (primary pancreatic tumour) | *CEBPA* | Missense mutation R86P | Non-synonymous coding | Targeted-NGS (MSK-IMPACT) | Surgical specimen | FFPE-(DS) |
|  | *ALK* | Fusion gene (partner: *STRN*) | Protein fusion: in frame (STRN-ALK) |  |  |  |
| Basturk, 2017 (recurrent pancreatic tumour) | *CDKN2A* | Nonsense mutation Y129* |  | Targeted-NGS (MSK-IMPACT) | Surgical specimen | FFPE-(DS) |
|  | *RET* | Missense mutation L80R | Non-synonymous coding |  |  |  |
|  | *ALK* | Fusion gene (partner: *STRN*) | Protein fusion: in frame (STRN-ALK) |  |  |  |
| Basturk, 2017 (celiac LN metastasis) | *ALK* | Fusion gene (partner: *STRN*) | Protein fusion: in frame (STRN-ALK) | Targeted-NGS (MSK-IMPACT) | Surgical specimen | FFPE-(DS) |
| Basturk, 2017 | *TRIP11* | Missense mutation L872H | Non-synonymous coding | Targeted-NGS (Ion Ampliseq) | Surgical specimen | Frozen tissue |
| Basturk, 2017 | *AXL* | Missense mutation R190H | Non-synonymous coding | Targeted-NGS (Ion Ampliseq) | Surgical specimen | Frozen tissue |
|  | *PIK3CB* | Missense mutation L35V | Non-synonymous coding |  |  |  |
| Basturk, 2017 | *ARHGAP35* | Nonsense mutation S975* |  | WES (SOLiD System) | Surgical specimen | Frozen tissue |
|  | *CBLB* | Missense mutation G259V | Non-synonymous coding |  |  |  |
|  | *CDKN2A* | Loss of expression |  |  |  |  |
|  | *CLK3* | Nonsense Mutation p.Y36* |  |  |  |  |
|  | *CNTN1* | Missense mutation P271L | Non-synonymous coding |  |  |  |
|  | *CTNNB1* | Missense mutation S45F | Non-synonymous coding |  |  |  |
|  | *EHBP1L1* | Missense mutation R1138H | Non-synonymous coding |  |  |  |
|  | *EPHB3* | Missense mutation Y855H | Non-synonymous coding |  |  |  |
|  | *ETFDH* | Missense mutation G75D | Non-synonymous coding |  |  |  |
|  | *FAM170A* | Missense mutation R65C | Non-synonymous coding |  |  |  |
|  | *FAM71E2* | Missense mutation L329M | Non-synonymous coding |  |  |  |
|  | *FAT1* | Missense mutation E2401K | Non-synonymous coding |  |  |  |
|  | *HIST1H4K* | Nonsense Mutation E64* |  |  |  |  |
|  | *JMJD6* | Missense mutation R95G | Non-synonymous coding |  |  |  |
|  | *KCNA5* | Missense mutation A50V | Non-synonymous coding |  |  |  |
|  | *KRT26* | Missense mutation R93C | Non-synonymous coding |  |  |  |
|  | *LTBP4* | Missense mutation G283D | Non-synonymous coding |  |  |  |
|  | *MCL* | Amplification |  |  |  |  |
|  | *MUC12* | Missense mutation N4428D | Non-synonymous coding |  |  |  |
|  | *MYB* | Missense mutation R73Q | Non-synonymous coding |  |  |  |
|  | *MYH13* | Missense mutation G203R | Non-synonymous coding |  |  |  |
|  | *MYH8* | Missense mutation R1715H | Non-synonymous coding |  |  |  |
|  | *NOL4* | Missense mutation T119M | Non-synonymous coding |  |  |  |
|  | *OSBPL8* | Missense mutation R318Q | Non-synonymous coding |  |  |  |
|  | *PIK3CA* | Missense mutation E545K | Non-synonymous coding |  |  |  |
|  | *SLC4A10* | Missense mutation D208H | Non-synonymous coding |  |  |  |
|  | *SYCP1* | Splice site mutation |  |  |  |  |
|  | *TM7SF3* | Frameshift Insertion S246fs |  |  |  |  |
|  | *TP53* | Missense mutation P113L | Non-synonymous coding |  |  |  |
|  | *UBASH3B* | Missense mutation E257K | Non-synonymous coding |  |  |  |
|  | *USH2A* | Missense mutation R878C | Non-synonymous coding |  |  |  |
| Basturk, 2017 | *APC* | Missense mutation A2T | Non-synonymous coding | WES (SOLiD System) | Surgical specimen | Frozen tissue |
|  | *CDKN2A* | Loss of expression |  |  |  |  |
|  | *CHML* | Missense mutation D210Y | Non-synonymous coding |  |  |  |
|  | *COL6A6* | Nonsense mutation R1502H |  |  |  |  |
|  | *CYR61* | Missense mutation C39* | Non-synonymous coding |  |  |  |
|  | *DNASE1L2* | Missense mutation S82F | Non-synonymous coding |  |  |  |
|  | *HIST1H3G* | Missense mutation M448K | Non-synonymous coding |  |  |  |
|  | *KALRN* | Missense mutation A364T | Non-synonymous coding |  |  |  |
|  | *KRAS* | Missense mutation S286T | Non-synonymous coding |  |  |  |
|  | *MCL* | Amplification |  |  |  |  |
|  | *MUC2* | Missense mutation A59E | Non-synonymous coding |  |  |  |
|  | *NF1* | Missense mutation K1111T | Non-synonymous coding |  |  |  |
|  | *PRR14L* | Nonsense mutation E658* |  |  |  |  |
|  | *SCN9A* | Missense mutation S1594T | Non-synonymous coding |  |  |  |
|  | *WRN* | Missense mutation E226K | Non-synonymous coding |  |  |  |
|  | *ZNF208* | Missense mutation V30L | Non-synonymous coding |  |  |  |
| Umemura, 2017 | none detected (tested genes: *TP53, PIK3CA, KRAS, BRAF, GNAS*) | - | - | NA | Surgical specimen | NA |
| Umemura, 2019* | none detected (tested genes: *TP53, PIK3CA, KRAS, BRAF, GNAS*) | - | - | NA | Surgical specimen | FFPE-(LMD) |
| Saeki, 2018 | none detected (tested genes: *PIK3CA, KRAS, BRAF, GNAS*) | - | - | NA | Surgical specimen | NA |
| Saeki, 2018* | none detected (tested genes: *PIK3CA, KRAS, BRAF, GNAS*) | - | - | NA | Surgical specimen | NA |

Abbreviations: ITPN, intraductal tubulopapillary neoplasm; LN: lymph node; NGS: next-generation sequencing; WGS: whole genome sequencing; WES: whole-exome sequencing FFPE: formalin-fixed paraffin-embedded tissue; FFPE-(DS): microdissection (not otherwise specified) from FFPE tissue slides; FFPE-(MDS): manual microdissection from FFPE tissue slides; FFPE-(LMD): laser microdissection from FFPE tissue slides; FFPE-(NOS): DNA obtained from FFPE tissue, not otherwise specified; NA: not available. Notes: *relapse in the same patient.
